# Neonatal and Postneonatal Pulmonary Hypertension

**DOI:** 10.3390/children8020131

**Published:** 2021-02-11

**Authors:** Satyan Lakshminrusimha

**Affiliations:** Department of Pediatrics, UC Davis Children’s Hospital; University of California Davis, Sacramento, CA 95817, USA; slakshmi@ucdavis.edu; Tel.: +1-916-734-5178

**Keywords:** oxygen, nitric oxide, sildenafil, pulmonary vascular resistance

## Abstract

During transition at birth with ventilation of the lungs, pulmonary vascular resistance (PVR) decreases from high fetal values, leading to an 8 to 10-fold increase in pulmonary blood flow (Qp). In some infants, this transition does not occur, resulting in pulmonary hypertension (PH). In infants, PH can present as: (a) primary PH in term neonates (idiopathic), (b) PH secondary to lung disease or hypoplasia in term infants, (c) acute PH in preterm infants with respiratory distress syndrome (RDS), (d) chronic PH with bronchopulmonary dysplasia (BPD) in preterm infants and (e) post-neonatal PH. A hemodynamically significant patent ductus arteriosus (PDA) can exacerbate PH in preterm infants due to increased Qp. Pulmonary vein stenosis (PVS) can complicate BPD with PH. Diagnosis of PH is based on clinical features, echocardiography and, in some intractable cases, cardiac catheterization. Therapy of PH includes oxygen, invasive or non-invasive ventilation, correction of acidosis, surfactant and selective and non-selective pulmonary vasodilators such as inhaled nitric oxide and sildenafil, respectively. Early closure of a hemodynamically significant PDA has the potential to limit pulmonary vascular remodeling associated with BPD and PH. The role of thiamine in pathogenesis of PH is also discussed with the recent increase in thiamine-responsive acute pulmonary hypertension in early infancy. Recognition and prompt therapy of PH can prevent right ventricular dysfunction, uncoupling and failure.

## 1. Introduction

During the fetal period, the placenta serves as the organ of gas exchange with umbilical venous PO_2_ in the low 30s and umbilical arterial PO_2_ in the low to mid-20s (mmHg) [1]. This relative hypoxemia results in hypoxic pulmonary vasoconstriction (HPV) resulting in high pulmonary vascular resistance (PVR) and low pulmonary blood flow (Qp) to the fetal lung. During birth, ventilation of the lungs with air increases alveolar and arterial PO_2_, resulting in pulmonary vasodilation, a drop in PVR and an 8 to 10-fold increase in Qp. Failure to drop PVR at birth due to various causes, such as birth asphyxia, parenchymal lung disease (e.g., meconium aspiration syndrome (MAS) [2], pneumonia, respiratory distress syndrome (RDS), etc., can lead to pulmonary hypertension (PH) in the newborn [3]. In some infants, the transition can happen in a normal pattern but postnatal increase in PVR can result in post-neonatal PH. Such increase can be seen in preterm infants with bronchopulmonary dysplasia (BPD) [4]. This special issue of *Children* includes articles on pathogenesis and management of neonatal and postneonatal PH.

## 2. Pulmonary Hypertension in Term Infants

Approximately 0.2% of term and late-preterm infants present with labile hypoxemia, bidirectional or left-to-right shunt across the patent ductus arteriosus (PDA) and oval foramen (PFO) in the immediate newborn period. These infants often exhibit hypoxemic respiratory failure (HRF) [3]. Secondary to shunting across the PDA, many of these infants present with differential cyanosis with higher SpO_2_ in the preductal regions (right hand) compared to postductal (any foot). In a lamb model of asphyxia, MAS and PH, Lesneski et al. demonstrate an association between the presence of a preductal to postductal SpO_2_ gradient of ≥3% with a higher proportion (89%) of samples associated with bidirectional shunting at PDA [5]. More importantly, low preductal to postductal SpO_2_ gradient (<3%) was still associated with bidirectional shunting at PDA in 56% of samples. This finding demonstrates that the lack of a preductal–postductal SpO_2_ gradient in a term infant does not rule out PH. Bidirectional shunt can still occur at the PFO level and present without this preductal to postductal SpO_2_ gradient as well. This findings in this study emphasize the importance of getting an early echocardiogram in infants with hypoxemia and suspected PH.

Ventilation with supplemental oxygen is the mainstay of management of infants with PH. The optimal SpO_2_ and PaO_2_ targets in neonatal PH and the optimal site (preductal vs. postductal) for checking these targets is not known. Chandrasekaran et al. discuss the important role of alveolar PAO_2_ in hypoxic pulmonary vasoconstriction [6]. As alveolar PAO_2_ matches preductal PaO_2_ more than postductal PaO_2_, monitoring preductal saturation is considered to be of greater benefit while managing PH [7]. Current guidelines recommend preductal SpO_2_ in the low to mid-90s and to avoid preductal SpO_2_ ≥98% during management of acute neonatal PH [8].

## 3. Pulmonary Hypertension in Preterm Infants

Among preterm infants, PH can present during an early period (usually first week) associated with respiratory distress syndrome (RDS) or later associated with bronchopulmonary dysplasia (BPD) [4]. The use of inhaled nitric oxide (iNO) in preterm infants is controversial [9,10]. The NIH consensus statement saw no evidence supporting the use of iNO in preterm infants < 34 weeks of gestation [11]. This statement did suggest that some infants with PH and pulmonary hypoplasia may benefit but data available were limited. Lack of clinical trials addressing PH in preterm infants is a major concern. Baczinski et al. have shown that almost half of preterm infants with PH can show acute echocardiographic response and such a positive response is associated with survival [12]. However, there are few studies evaluating therapies other than iNO in preterm infants with BPD. 

**Figure 1:**
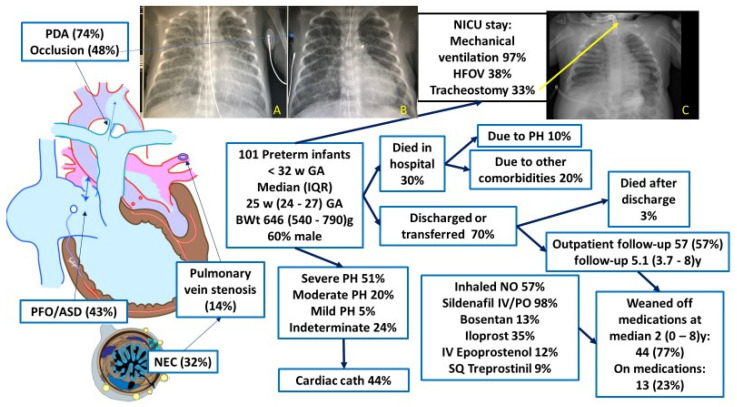
Graphic summary of Nees et al. [17] showing the course of preterm infants with bronchopulmonary dysplasia (BPD) and pulmonary hypertension (PH). The chest X-rays A and B show reduction in pulmonary vascularity after occlusion of the patent ductus arteriosus (PDA) by the transcatheter method. Chest X-ray C shows BPD with PH requiring a tracheostomy. Copyright Satyan Lakshminrusimha. Images courtesy Dr. Frank Ing.

Dr. William Northway identified and published the first report of BPD in 1967 [13]. The presence of PH along with BPD is associated with increased mortality in these patients and is directly related to severity of PH. Increased pulmonary vascular tone and reactivity, vascular remodeling, abnormal vasculogenesis and angiogenesis contribute to PH in BPD [4]. The risk factors for PH in preterm infants includes low birth weight, SGA status, severity of BPD, oligohydraminios, maternal smoking and maternal preeclampsia. Data from serial echocardiograms shows that early evidence of PH (septal flattening by echo on day 7 of postnatal age) may be associated with poor outcomes including increased mortality [14]. Interestingly, increased target SpO_2_ [15] and closure of atrial septal communications [16] may reduce the risk of PH in preterm infants. Therapeutic goals to enhance transition at birth, prevent BPD and reduce risk of PH, and eventually to improve long term outcomes, are important. In this issue of *Children*, Nees et al. present data on targeted therapy in 101 preterm infants with BPD and PH (Figure 1). Although the mortality rate was high (32.7%), few deaths occurred after hospital discharge and 77.2% of patients at follow-up were weaned off PH medications by a median of 2 years (range 0–8 y) [17].

## 4. Role of the PDA

**Figure 2:**
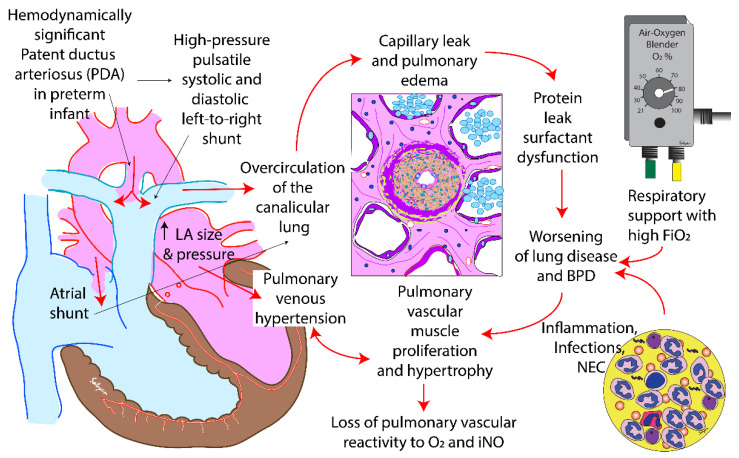
The impact of a hemodynamically significant patent ductus arteriosus (PDA) in preterm infants. The continuous flow from left-to-right leads to pulmonary overcirculation, protein leak and pulmonary edema. Oxygen free radical injury, volutrauma and barotrauma induced by ventilation and inflammation caused by recurrent infections and necrotizing enterocolitis (NEC) can exacerbate lung injury leading to bronchopulmonary dysplasia (BPD). Pulmonary vascular remodeling can be associated with poor response to oxygen and inhaled nitric oxide (iNO). Prolonged increased in pulmonary blood flow increases the size and pressure in the left atrium (LA) and can eventually lead to pulmonary venous hypertension. Copyright Satyan Lakshminrusimha.

Very low birth weight infants have an open ductus in the first few days after birth. In the majority of these infants, the ductus gradually closes with postnatal age. However, in less mature babies (<26 weeks gestation at birth), the ductus is likely to remain open for longer periods of time. Persistently open PDA and pulmonary over-circulation will eventually lead to increased left atrial pressure and left ventricular diastolic dysfunction (Figure 2). 

**Figure 3:**
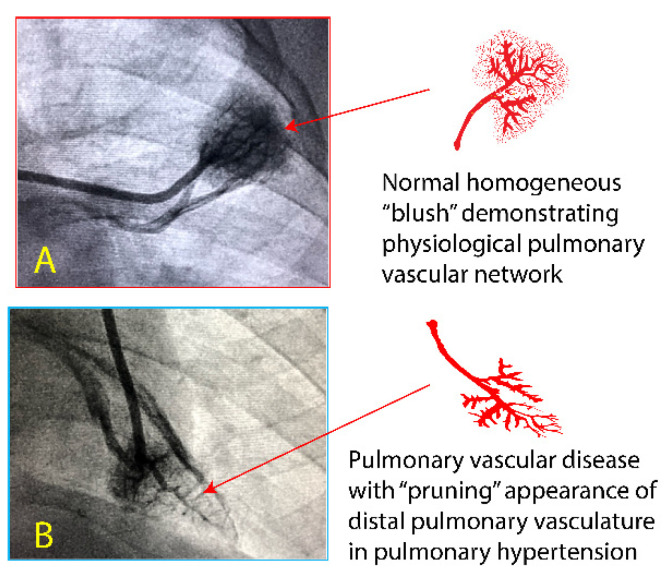
The pulmonary vascular changes observed during cardiac catheterization in preterm infants. (**A**). Normal pulmonary vasculature with nice arborization of pulmonary arterial tree is seen as a “blush”. (**B**). Remodeled pulmonary vascular network leads to a “pruning” or “moth-eaten” appearance where distal vessels are lost. Cardiac catherization images courtesy Professor Frank Ing, Chief of Pediatric Cardiology, UC Davis Children’s Hospital (with permission). Copyright Frank Ing and Satyan Lakshminrusimha.

Extremely preterm infants < 25 weeks, <750 grams birth weight with massive left-to-right shunt due to PDA, might be at risk of pulmonary vascular remodeling (Figure 3). This remodeling can be recognized during cardiac catheterization by the appearance of distal pulmonary vasculature. Pulmonary vascular disease may lead to right ventricular dysfunction. Delay in closure of PDA is associated with increased PVR [18]. Extremely preterm infants with persistent elevation of respiratory severity score after PDA closure appear to have two risk factors: high PVR prior to closure and prolonged exposure to PDA (>8 weeks). Does transcatheter closure of PDA in preterm infants decrease the risk of pulmonary hypertension in these infants? Is the benefit of transcutaneous closure limited to infants requiring significant respiratory support such as invasive mechanical ventilation? These questions are being addressed by the Preliminary Percutaneous Intervention vs. Observational Trial of Arterial Ductus in Low-weight infants (PIVOTAL) (NCT03982342). The risk of anesthesia and catheterization must be considered while making a decision about transcatheter closure of PDA [19].

**Figure 4:**
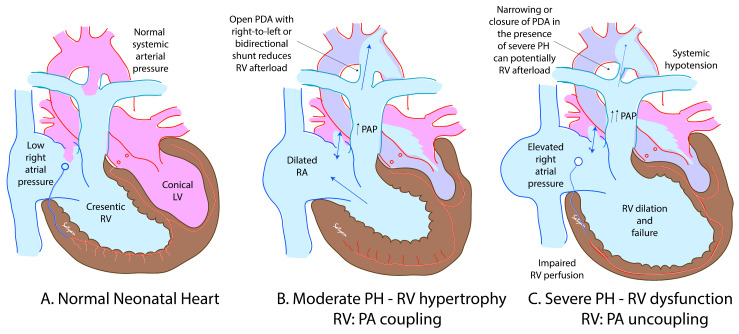
The relationship between right ventricular (RV) function and afterload (pulmonary arterial pressure—PAP). In normal neonates, PAP is low (**A**). The left ventricle (LV) is conical in shape and can work efficiently against high afterload. On the other hand, RV is crescentic in shape and does not handle severe increases in afterload. With mild to moderate elevation in PAP, compensatory RV hypertrophy with enhanced function is observed (**B**). However, with persistent elevation of PAP, RV dysfunction and failure set in. A closing ductus can remove the right ventricular afterload pop-off and contribute to RV dysfunction. High right atrial pressure and borderline low systemic blood pressure can impair coronary perfusion and further reduce RV function. The presence of a right-to-left shunt across the PDA (**C**) can reduce afterload. Copyright Satyan Lakshminrusimha.

In normal neonates, the left ventricle is conical in shape and contracts efficiently against high afterload (systemic pressure) as shown in Figure 4A. The right ventricle is crescentic and typically is exposed to low afterload (pulmonary arterial pressure). Increasing pulmonary arterial pressure increases right ventricular afterload and initially increases the efficiency of the right ventricle by enhancing function and causing right ventricular hypertrophy by a phenomenon known as RV:PA coupling (Figure 4B). The presence of an open ductus functions as a pop-off valve and limits extreme elevations in pulmonary arterial pressures. However, extreme elevation in afterload, often associated with closing ductus can uncouple the right ventricle leading to dysfunction (Figure 4C).

## 5. Postneonatal Pulmonary Hypertension and Thiamine Deficiency

After the neonatal period, in the absence of BPD or other lung conditions, PH is uncommon. More recently, there have been several reports of acute PH in early infancy [20]. These infants demonstrate rapid deterioration with high mortality without specific therapy. Treatment with thiamine has led to a dramatic improvement in PH. Public education and appropriate supplementation of dietary thiamine among breastfeeding mothers are important to prevent this condition. 

## 6. Discussion

Pulmonary hypertension during neonatal and post-neonatal period continues to contribute to morbidity and mortality. Early recognition and targeted therapy of PH can lead to improvement in survival. This issue of *Children* includes several papers by eminent authors addressing some of the controversies in diagnosis and management of neonatal and postneonatal PH.

## Data Availability

Data sharing not applicable. No new data were created or analyzed in this study. Data sharing is not applicable to this article.
